# Targeted deep sequencing of mycobacteria species from extrapulmonary sites not identified by routine line probe assays: A retrospective laboratory analysis of stored clinical cultures

**DOI:** 10.1016/j.ijregi.2024.100464

**Published:** 2024-09-24

**Authors:** Christoffel Opperman, Janré Steyn, Megan Ceris Matthews, Sarishna Singh, Yonas Ghebrekristos, Tanya Jane Kerr, Michele Miller, Aliasgar Esmail, Helen Cox, Robin Warren, Giovanni Ghielmetti, Wynand Goosen

**Affiliations:** 1 National Health Laboratory Service, Green Point TB-Laboratory, Cape Town, South Africa; 2 SAMRC Centre for Tuberculosis Research, Division of Molecular Biology and Human Genetics, Stellenbosch University, Cape Town, South Africa; 3 Division of Medical Microbiology, Department of Pathology, University of Cape Town, Cape Town, South Africa; 4 UCT Lung Institute, Centre for Lung Infection and Immunity. Division of Pulmonology, Department of Medicine, University of Cape Town and Groote Schuur Hospital, Cape Town, South Africa; 5 Division of Medical Microbiology, Department of Pathology and the Institute of Infectious Disease and Molecular Medicine and Wellcome Centre for Infectious Disease Research, University of Cape Town, Cape Town, South Africa; 6 Section of Veterinary Bacteriology, Institute for Food Safety and Hygiene, Vetsuisse Faculty, University of Zurich, Zurich, Switzerland; 7 Department of Microbiology and Biochemistry, University of the Free State, Bloemfontein, South Africa

**Keywords:** Nontuberculous mycobacteria, Mycobacteria, Oxford nanopore technologies sequencing, Targeted amplicon sequencing, Co-infection, Extrapulmonary

## Abstract

**Objectives:**

Nontuberculous mycobacteria (NTM) infections present a global health challenge. This study describes unidentified mycobacteria species from extrapulmonary sites, using advanced identification and sequencing techniques.

**Methods:**

Extrapulmonary mycobacteria growth indicator tube primary cultures collected retrospectively between 2019 and 2023, featuring unidentified mycobacteria species detected by GenoType Mycobacterium line probe assays, underwent multilocus targeted next-generation sequencing using Oxford Nanopore Technology, polymerase chain reaction amplicon Sanger sequencing, and Deeplex Myc-TB analysis. Previously collected clinical and laboratory data were reported.

**Results:**

A total of 28 cultures, collected from extrapulmonary sites, each from different patients, were included. Mycobacterial mixtures were identified in 19 of 28 (68%) cultures, with four of 28 (14%) showing unidentified species based on sequencing of *rpoB* and *hsp65* targets. *Mycobacterium monacense* was present in 13 of 28 (46%) of the cultures. Culturable *Mycobacterium tuberculosis* complex (MTBC) was identified in five extrapulmonary specimens that previously tested negative for MTBC using Xpert MTB/RIF Ultra. The comparative analysis between Sanger and targeted next-generation sequencing using Oxford Nanopore Technology sequencing (for *hsp65*) demonstrated 27 of 28 (96%) agreement on the predominant strain. Deeplex Myc-TB could not identify NTM-MTBC co-infections in minor subpopulations.

**Conclusions:**

This study highlights the role of advanced sequencing in identifying NTM mixtures and mycobacterial co-infections. It calls for ongoing efforts to integrate next-generation sequencing into mycobacteria testing algorithms.

## Highlights

• Deep sequencing identifies nontuberculous mycobacteria (NTM) subpopulation variants.
• *Mycobacterium monacense* is a prominent NTM among extrapulmonary sites.
• Deep sequencing confirms *M. tuberculosis* complex (MTBC) in Xpert MTB/RIF Ultra–negative cultures.
• Deep sequencing can detect NTM mixtures and MTBC co-infection.
• Oxford Nanopore Technology sequencing is superior to Deeplex Myc-TB in detecting MTBC.

## Introduction

Nontuberculous mycobacterial (NTM) infections, which are often neglected, are now emerging as a substantial global health challenge [1]. Ubiquitously found in water and soil, NTMs are commonly considered contaminants and are generally perceived as opportunistic pathogens [2]. Approximately 30 of more than 200 known NTM species are recognized as pathogenic [3]. Examples include *M. monacense*, reported to cause conditions such as osteomyelitis, bacteremia, and ascites [4,5], whereas *M. novocastrense* has been associated with pulmonary and wound infections [6]. Some NTM, such as *M. sherrisii*, are known to mimic gynecological malignancies [7]. Several other mycobacterial species, including *M. holsaticum, M. perigrinum, M. insubricum, M. kyorinense*, and *M. duvalli*, have been recognized as causative agents of diverse extrapulmonary diseases [8]. Current clinical algorithms in high *Mycobacterium tuberculosis* complex (MTBC) settings are focused on tuberculosis and disregard NTM infections [9]. Therefore, the identification of clinically relevant NTMs for further investigation remains crucial. Improving our ability to identify and characterize NTMs would enhance our understanding of which NTM species pose the greatest threat to human health.

The advent of next-generation sequencing (NGS) tools has facilitated the identification of more than one NTM or NTM together with other mycobacteria from a single culture (collectively referred to as NTM mixtures in this investigation) [10,11]. Recent findings indicate that line probe assay (LPA) techniques may fail to recognize NTM mixtures and, consequently, necessitate pure cultures for NTM identification [12]. The application of polymerase chain reaction (PCR) and sequencing methods, specifically, the concurrent analysis of different target gene sequences such as RNA (ribonucleic acid) polymerase beta subunit (*rpoB*) and heat-shock protein 65 (*hsp65*), has proved to be instrumental in the identification of NTM mixtures [13]. This approach augments our ability to discern diverse NTM species within complex cultures.

The utilization of Deeplex Myc-TB targeted NGS assay (Genoscreen, Lille, France) has emerged as a promising modality for the identification of MTBC [14]. The Deeplex Myc-TB assay is an amplicon-based deep sequencing platform also targeting *hsp65*, capable of detecting as low as 3% of the minority strains in heteroresistant cultures [15]. In addition to MTBC, the Deeplex Myc-TB can identify >100 different NTM species [1]. Therefore, as future studies explore treatment regimens and clinical outcomes associated with mixed infections involving different mycobacterial species, the expansion and enhancement of accurate diagnostic detection platforms is paramount.

To describe the presence of potentially clinically relevant mycobacterial species, we used a range of sequencing approaches on stored extrapulmonary clinical cultures.

## Methods

### Setting

The National Health Laboratory Service, TB laboratory in Cape Town, is a public diagnostic referral laboratory for the Western Cape Department of Health and Cape Town, City Health, South Africa. On average, it performs 60,000 TB-related tests per month. It receives cultures from 53 hospitals and 170 primary health care facilities in and around Cape Town. The laboratory serves a Western Cape population of approximately 7,433,020 [16]. Among the extrapulmonary isolates reported in 2020 (Figure S1), the majority were unidentified *Mycobacterium* spp., accounting for 40% (32 of 80) of the total isolates. This was followed by members of the *Mycobacterium avium* complex, with *M. avium* comprising 32.5% (26 of 80) and *Mycobacterium intracellulare* 15% (12 of 80). The most prevalent condition associated with these isolates was bacteremia, occurring in 37.5% (30 of 80) of cases.

### Study design

**Figure 1:**
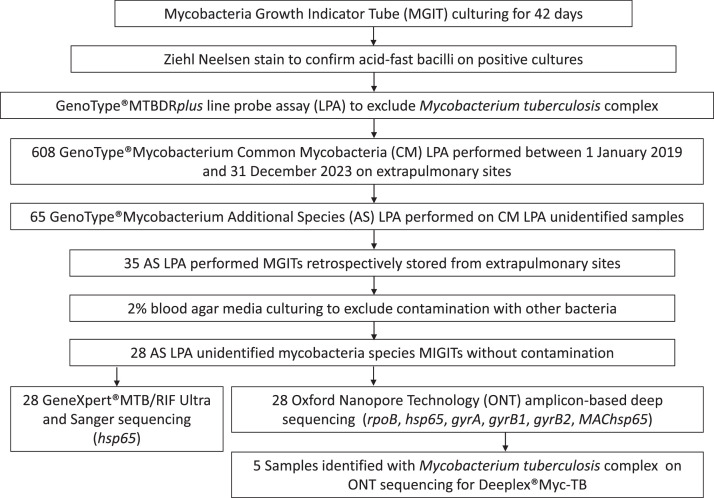
Retrospective screening of mycobacteria cultures for unidentified species from clinical extrapulmonary sites across the Western Cape Province, South Africa, conducted between 1 January 2019 and 31 December 2023. The routine laboratory workflow included the GenoType MTBDR*plus* line probe assay (LPA), GenoType Mycobacterium Common Mycobacteria (CM) and Additional Species (AS) LPA. The AS LPA was only performed if the culture originated from an extrapulmonary site, if NTM was repeatedly detected in pulmonary cultures, or if specifically requested by the attending clinician or TB pathologist reviewing the results. Twenty-eight stored cultures without contamination were available for investigation. Study-specific methodology was employed on all 28 positive Mycobacterium Growth Indicator Tubes (MGITs) in the culture cohort, including the use of Xpert MTB/RIF Ultra to exclude *Mycobacterium tuberculosis* complex (MTBC) and 2% blood agar to eliminate contamination with other bacteria. All 28 available cultures were subjected to targeted amplicon-based deep sequencing (ONT, Oxford Nanopore Technology) and Sanger sequencing (*hsp65*) analysis. Deeplex Myc-TB was performed on all cultures identified with MTBC from ONT sequencing. *rpoB,* RNA polymerase beta subunit, *hsp65*, heat-shock protein 65, *gyrA,* DNA gyrase A, *gyrB1,* DNA gyrase B1, *gyrB2,* DNA gyrase B2, *MAChsp65, Mycobacterium avium* complex heat-shock protein 65.

A total of 608 extrapulmonary cultures, subjected to GenoType Mycobacterium Common Mycobacteria (CM) LPA testing between 1 January 2019 and 31 December 2023, were retrospectively screened for possible inclusion. From these, 65 exhibited mycobacteria unidentified to the species level on GenoType Mycobacterium CM and Additional Species (AS) LPA (Bruker, Billerica, Massachusetts, USA). Primary mycobacteria growth indicator tube (MGIT) (Becton Dickinson, Berkshire, United Kingdom) cultures without contamination (bacteria other than mycobacteria) on 2% blood agar (Merck, Darmstadt, Germany) were available for 28 clinical cultures collected from extrapulmonary sites, out of a possible 35 stored cultures (20% contaminated). Contaminated MGITs were excluded to avoid including cultures where other organisms might inhibit or overgrow the mycobacteria or where breaches in standard operating procedures could have introduced NTM during processing. Sanger sequencing and targeted next-generation long-read sequencing (tNGS) with Oxford Nanopore Technology (ONT) (Oxford, United Kingdom) were performed on all 28 cultures. All cultures that indicated MTBC on the ONT sequencing underwent Deeplex Myc-TB targeted NGS analysis (Figure 1).

### Laboratory methods

Routine laboratory processes included a Ziehl-Neelsen stain on all positive MGIT cultures to confirm the presence of acid-fast bacilli after a 42-day incubation period. The dominant presence of acid-fast bacilli after MGIT culturing ensures the abundance of viable mycobacteria. The GenoType MTBDR*plus* LPA (Bruker, Billerica, Massachusetts, USA) initially excluded MTBC (afterward identified with tNGS ONT sequencing in the same cultures), and a subsequent GenoType Mycobacterium CM and AS LPA could not identify the *Mycobacterium* spp*.* In addition to routine laboratory methods to exclude MTBC, the Xpert MTB/RIF Ultra assay (GXPU) (Cepheid, Sunnyvale, California, USA) performed on the positive stored MGIT culture confirmed the absence of insertion segments (IS*1081* and IS*6110*), i.e., MTBC (Figure 1).

### Ethics statement

This study received ethical approval from the human research ethics committee of Stellenbosch University (reference number: S22/10/191). In addition, repository collection approval for the storage of rare NTM cultures was obtained from the University of Cape Town (reference number: R013/2023). Institutional approval was granted by the National Health Laboratory Service (PR2232714).

### Clinical and laboratory data

Laboratory results and clinical data, available on the laboratory information system (TrakCare, Version L2016, InterSystems, Sydney, Australia), were reviewed to infer the possible significance of the MGIT cultured mycobacteria. In addition, 1-year all-cause mortality was assessed via Clinicom, a single shared hospital patient administration system.

### Sanger sequencing

To identify mycobacterial species present, PCR amplification of *hsp65* was performed according to Clarke et al. [17], after which a comparison between Sanger sequencing and targeted amplicon ONT sequencing was performed. Amplicons were forwarded to the Central Analytical Facility at Stellenbosch University, Cape Town, South Africa for Sanger sequencing. Pairwise sequence alignments were subsequently conducted using A Plasmid Editor (v3.1.3) [18]. The consensus sequences were subjected to analysis through the National Centre for Biotechnology Information nucleotide Basic Local Alignment Search Tool software program [19]. A stringent similarity index and gene coverage of ≥98% was deemed accurate for identification, aligning with the type strain in GenBank (http://www.ncbi.nlm.nih.gov/genbank/).

### Targeted NGS using Oxford nanopore technologies sequencing platform

Extracted DNA was used to amplify *rpoB, hsp65*, DNA gyrase A (*gyrA*), DNA gyrase B1 (*gyrB1*), DNA gyrase B2 (*gyrB2*), and *M. avium* complex heat-shock protein 65 (MAC*hsp65*) gene regions. The primers, thermocycler conditions, and expected product sizes are available in the supplementary material Table S1. Amplified PCR products were visualized with 1.5% agarose gel electrophoresis. To ascertain DNA concentrations of PCR amplicons, the Qubit double-stranded (ds) DNA high sensitivity (HS) Assay kit (Life Technologies, California, USA) was used, adhering to the manufacturer's instructions. After this, the amplicons underwent end repair (NEB Next Ultra II End Repair/dA Tailing Module; New England Biolabs, Massachusetts, USA). Amplicons within the same culture were barcoded using an ONT Native Barcoding Kit v14 (ONT, Oxford, UK) and combined in equal molar concentrations (300 femtomole) into a unified library. Libraries were ligated with native adapters (NEB Blunt/TA Ligase Master Mix and Quick Ligation Module) and loaded onto a single R10.4.1 flow cell (>1350 active pores; ONT). The sequencing was carried out using the MinION mk1C device (ONT). Additional information on the in-house developed tNGS ONT long-read sequencing method is available in the supplementary material.

### Bioinformatics for targeted NGS using Oxford nanopore technologies sequencing

Bioinformatics analysis followed the approach outlined by Ghielmetti et al. [20]. In summary, 28 data sets generated by sequencing carried out using the MinION mk1C device were subjected to analysis. Base calling, de-multiplexing, and barcode trimming were performed using Guppy (v6.4.6) after exceeding 24 hours of data acquisition. The quality assessment of sequencing reads involved the use of FastQC (v0.11.9) and pycoQC (v2.5.0.23). Nanoq (v0.10.0) was used for filtering reads with a Q score below 12 and generating summary reports. Consensus sequences were grouped based on amplicon size and genetic similarity and relative abundances retrieved based on the representative pool of reads analyzed. The ABRicate software tool was applied for the screening of consensus sequences against customized databases for each target and generate summarizing report files [20,21]. The supplementary material provides details on the reference-free read sorting, nomenclature used, coverage, and identity match criteria used for classification.

### Phylogenetic analysis of targeted amplicon-based NGS data

Consensus sequences that could not be unequivocally identified at the species level were included in two phylogenetic trees comprising 119 and 195 reference sequences for *hsp65* and *rpoB*, respectively. Briefly, the variable single nucleotide polymorphism alignments of the consensus sequences and the corresponding references were used to infer maximum likelihood phylogeny using the neighbor-joining method [22], bootstrapped 100 times, supported by the CLC Genomics Workbench 23.0.5 (Qiagen), and visualized using Interactive Tree of Life [23].

### Deeplex Myc-TB analysis

All five isolates (Table 2) that displayed MTBC-specific variants during tNGS ONT deep sequencing were included for Deeplex Myc-TB targeted NGS analysis to confirm the presence of MTBC. Extracted DNA was subjected to PCR, tagmentation, and library preparation using Illumina DNA reagents (Illumina, San Diego, California, USA) according to manufacturer requirements. A detailed account of the Deeplex Myc-TB procedure can be obtained in the supplementary material under Deeplex Myc-TB analysis. Post-amplicon cleanup included Agencourt AMPure XP (Beckman-Coulter, California, USA) magnetic bead separation. For tagmentation, a total of 100 nanograms of the PCR products were added into a 50 µL reaction containing bead-linked transposomes and tagmentation buffer. Tagmented libraries underwent further purification and quantification using Qubit dsDNA HS Assay and were pooled. PhiX denaturation preceded library dilution, and the final loading volume for the MiniSeq System (Illumina, San Diego, California, USA) was 500 µl at a concentration of 1.2 picomolar with a 1% PhiX spike. The FASTq files were analyzed using the Deeplex Myc-TB V3_0_1 Extended catalog on the Genoscreen Deeplex Myc-TB online application.

## Results

### Clinical and laboratory data

A total of 28 MGIT primary cultures from extrapulmonary clinical sites were included (Table 1). The average time to MGIT culture positivity was 18.9 days (range 6-40). All the MGIT cultures were confirmed to be mycobacteria species other than MTBC with the GenoType Mycobacterium CM and AS LPAs. These cultures comprised fluid aspirates (n = 15 of 28; 53.6%) from different anatomical sites, cerebrospinal fluids (n = five of 28; 17.9%), fine-needle aspirations (n = four of 28; 14.3%), urine (n = two of 28; 7.1%), and pus aspirates (n = two of 28; 7.1%). Among the patients, seven (seven of 28; 25%) experienced all-cause mortality within 1 year. Most of the patients were female (15 of 28, 53.6%), with a cohort median age of 44.5 years (interquartile range: 32.5-53.5). A total of 12 patients (12 of 28; 42.9%) tested positive for HIV (cluster of differentiation 4+ T-cell range: 4-732), and five (five of 28; 17.9%) recently received confirmation of MTBC infection with a positive GXPU sputum result (within 3 weeks before or after the NTM culture).

**Table 1:** Integration of clinical and laboratory characteristics, with sequencing results for liquid cultures harboring unidentified nontuberculous mycobacteria species according to line probe assays.

Patient culture	Clinical culture type (site)^a^	Collection year	Age (years)/Sex	Working diagnosis/Underlying comorbidities^b^	Clinical outcome^c^	TPP	Laboratory results	HIV status^d^ (Absolute CD4 count)	Previous MTBC confirmed^e^ (Site)^a^	Sanger sequencing of heat-shock protein 65 (*hsp65*) gene from Mycobacteria growth indicator tubes			tNGS ONT Predominant species	Mixed according to tNGS ONT^f^
										Percentage cover of gene (%) /Expect value^g^	Percentage identity (%)^h^	*Mycobacterium species*	Predominant species on *hsp65* (Percentage)	
1	Fluid aspirate (pleural)	2021	23/F	Pneumonia with a pleural effusion	Alive	25	Fluid exudate^i^/ No other bacteria cultured on the fluid	N	Y (sputum)	100	97.1	*Mycobacteria spp.*	*Mycobacteria spp.* (100)	UMS^j^
2	FNA: Fine needle aspiration	2021	40/F	Malignancy/HPT, DMII	Alive	8	Histology normal with no malignancy	P (79)	N	100	99.1	*M. monacense*	*M. monacense* (100)	Yes
3	CSF	2021	15/F	Meningitis	Alive	15	CSF acellular with a normal glucose and protein	N	N	100	100	*M. duvalii*	*M. duvalii* (97.2)	Yes
4	Synovial fluid (Knee)	2021	50/M	Synovitis/Bursitis	Alive	10	No other bacteria cultured on the fluid	N	N	100	98.6	*M. monacense*	*M. monacense* (100)	No
5	Fluid aspirate (left knee)	2021	48/M	Septic arthritis	Alive	11	No other bacteria cultured on the fluid	P (406)	N	98	93.1	*Mycobacteria spp.*	*Mycobacteria spp.* (100)	Yes
6	Urine	2019	35/M	Cystitis (uncomplicated urinary tract infection)	Alive	10	Urine: 180,000 leucocytes/milliliter and cultured *Morganella morganii*	U	N	100	98.8	*M. malmesburyense*	*M. malmesburyense* (96.1)	Yes
7	Fluid aspirate (Ascitic)	2020	31/M	Liver impairment/Jaundice	Deceased	9	Cytology: Histiocytic effusion/Hepatitis B and C markers negative	U	N	100	98.6	*M. elephantis*	*M. elephantis* (70.3)	Yes
8	CSF	2022	34/M	Meningitis	Alive	23	CSF: Lymphocytes (3) with a normal glucose and protein	P (18)	Y (sputum)	100	98.6	*M. holsaticum*	*M. holsaticum* (99.4)	Yes
9	Fluid aspirate (pleural)	2020	80/M	Pleural effusion/AF on warfarin, CKD, HPT	Alive	17	Fluid transudate^i^/No other bacteria cultured on the fluid	N	N	100	98.1	*M. monacense*	*M. monacense* (100)	Yes
10	CSF	2019	46/F	Meningitis/Sepsis of unknown source	Deceased	40	CSF acellular with a normal glucose and protein	Y (4)	N	96	99.1	*Mycobacteria spp.*	*Mycobacteria spp.* (100)	Yes
11	Synovial fluid (Knee)	2020	28/M	Septic arthritis/Ankylosing spondylitis	Alive	36	*Staphylococcus aureus* cultured from the knee fluid	N	N	99	99.3	*M. monacense*	*M. monacense* (100)	No
12	Fluid aspirate (peritoneal)	2020	53/M	Malignancy/Tuberculosis	Alive	14	Fluid exudate^i^/ Cytology: No malignancy with scattered lymphocytes	Y (223)	N	100	100	*M. monacense*	*M. monacense* (100)	No
13	Pleural fluid (right lung)	2020	24/F	Sepsis due to complicated lower respiratory tract infection	Alive	30	Fluid exudate^i^/No other bacteria cultured on the fluid	N	Y (sputum)	100	99.4	*M. novocastrense*	*M. novocastrense* (100)	Yes
14	Pus (left Lung)	2019	54/F	Empyema	Alive	15	Fluid exudate^i^/No other bacteria cultured on the pus	Y (732)	Y (sputum)	100	99.7	*M. monacense*	*M. monacense* (100)	No
15	Pleural fluid (right lung)	2019	49/F	COPD, Cor-pulmonale	Deceased	15	Fluid exudate^i^/No other bacteria cultured on the fluid	U	N	100	94.7	*Mycobacteria spp.*	*Mycobacteria spp.* (100)	UMS
16	Pleural fluid (right lung)	2019	80/F	Pulmonary malignancy	Deceased	35	Cytology: Adenocarcinoma	U	N	100	99.1	*M. gallinarum*	*M. gallinarum (100)*	Yes
17	Urine	2022	41/F	Uncomplicated urinary tract infection	Alive	39	Urine: Abundant leucocytes with erythrocytes/*Escherichia coli* cultured	Y (108)	N	100	98.3	*M. brumae*	*M. brumae* (97.3)	Yes
18	CSF	2021	43/F	Meningitis	Alive	14	CSF: Neutrophils (5), lymphocytes (8), protein raised with a normal glucose	Y (345)	N	97	98	*Mycobacteria spp.*	*Mycobacteria spp.* (100)	UMS
19	Pleural fluid (right lung)	2021	73/M	Pneumonia/Malignancy	Deceased	21	Histology: Desmoplastic mesothelioma	N	N	100	98.8	*M. novocastrense*	*M. novocastrense* (100)	Yes
20	Fluid aspirate (right neck mass)	2022	29/M	Tuberculosis	Alive	9	FNA: Sydney III-Atypical cells	U	N	100	99.4	*M. malmesburyense*	*M. malmesburyense* (96.6)	Yes
21	CSF	2022	58/F	Guillain-Barré syndrome	Alive	11	CSF: Lymphocytes (2), erythrocytes (6)/Syphilis and Cryptococcal antigen negative	N	N	100	99.1	*M. novocastrense*	*M. novocastrense* (98.1)	Yes
22	Fluid aspirate (peritoneal)	2022	47/F	Malignancy or tuberculosis/ DMII, HPT	Alive	9	Histology: Steroid cell tumor	Y (824)	N	99	99.4	*M. alvei*	*M. alvei* (94.4)	Yes
23	Fluid aspirate (right lung)	2022	87/M	Pneumonia with a pleural effusion	Deceased	21	Fluid exudate^i^/ No other bacteria cultured on the fluid	U	N	100	100	*M. avium*	*Mycobacteria spp.* (100)	Yes
24	Fine needle aspirate (right axillary lymph node)	2023	10/F	Generalized lymphadenopathy and weight loss	Alive	25	FNA: Reactive lymph node/No malignancy detected	Y (129)	N	99	98.5	*M. monacense*	*M. monacense* (100)	Yes
25	Pleural fluid (right lung)	2023	50/M	Pneumonia with a pleural effusion	Alive	12	Fluid exudate^i^/No other bacteria cultured on the fluid	U	Y (sputum)	100	99.7	*M. monacense*	*M. monacense* (100)	No
26	Pus aspirate (left inguinal mass)	2022	40/F	Malignancy or tuberculosis/DM II, HPT	Alive	6	No other bacteria cultured/No malignancy detected	P (233)	N	96	99.1	*Mycobacteria spp.*	*Mycobacteria spp.* (100)	UMS
27	FNA (lymph node)	2022	41/M	Malignancy or tuberculosis	Alive	30	Cytology: Atypical cells, no biopsy submitted	P (271)	N	100	95.9	*Mycobacteria spp.*	*Mycobacteria spp.* (99.1)	Yes
28	FNA (left axillary lymph node)	2023	68/F	Metastatic malignancy	Deceased	20	Histology: Metastatic breast adenocarcinoma	N	N	100	98	*M. monacense*	*M. monacense* (100)	Yes

CSF, cerebrospinal fluid; FNA, fine-needle aspirate; TTP, time to culture positivity in days; MTBC, *Mycobacterium tuberculosis* complex; ONT, Oxford nanopore technology; tNGS, targeted next-generation sequencing; UMS, uncharacterized mycobacteria species.

a Site indicated when provided on the laboratory request form.

b Co-morbidities are reported when available on the laboratory information system. AF- Arterial fibrillation; CKD: chronic kidney disease; COPD: chronic obstructive pulmonary disease; DMII: diabetes mellitus type 2; HPT: hypertension.

c One-year all-cause mortality.

d HIV is reported as P: positive, N: negative, U: untested.

e MTBC is reported as P: positive, N: negative.

f The interpretation of a mycobacteria mixture is based on tNGS ONT amplicon-based deep sequencing of multiple targets.

g The expect (E) value, in the context of Basic Local Alignment Search Tool (BLAST), represents the number of chance alignments anticipated to have the same score or a superior one purely by random occurrence. An E-value smaller than 1^e-50^ signifies matches within the database that are of exceptionally high quality and was found for all Sanger sequencing results.

h A stringent GenBank (National Institutes of Health genetic sequence database of publicly available DNA sequences) similarity alignment percentage and gene coverage of 98% was deemed accurate for identification.

i According to Light's biochemical criteria.

j The presence of uncharacterized mycobacteria species is based on the targets used for amplicon-based deep sequencing. As a result, making a final assumption regarding the presence of multiple mycobacteria is not possible.

### Sanger sequencing

Among the 28 sequenced cultures, *M. monacense* was detected in nine of 28 (32%) (Table 1). The *hsp65* gene exhibited a gene coverage and similarity alignment percentage exceeding ≥98% across 21 of 28 (75%) cultures (Table 1). This subset included representatives from various species, including *M. monacense* (n = nine of 28; 32.1%); *Mycobacteria* spp. (n = seven of 28; 25%); *M. novocastrense* (n = 3 of 28; 10.7%); *M. malmesburyense* (n = two of 28; 7.1%); and single isolates (n = one of 28; 3.6%) of *M. alvei, M. avium, M. brumae, M. duvalii, M. elephantis, M. gallinarum*, and *M. holsaticum*.

### Targeted NGS using Oxford nanopore technologies sequencing platform

More than 15 million reads were generated from the 28 cultures. All cultures exhibited a sequencing depth exceeding 100,000 reads and a mean read quality score greater than 14 (Figure S2). The *hsp65* gene successfully identified diverse mycobacterial species in various extrapulmonary sites, including members of the MTBC and several NTM species, namely, *M. novocastrense* (cultures 13 [100%], 19 [100%], 20 [0.3%], 21 [98.1%]), *M.* monacense (cultures 2 [100%], 3 [1.8%], 4 [100%], 9 [100%], 11 [100%], 12 [100%], 14 [100%], 17 [2.3%], 20 [1.2%], 22 [3.8%], 24 [100%], 25 [100%], 28 [100%]), *M. duvalii* (cultures 3 [97.2%]), *M. malmesburyense* (cultures 6 [96.1%], 8 [0.6%], 20 [96.6%]), *M. senuense* (culture 21 [1.6%]), *M. abscessus* subsp*.* b*olletii* (culture 7 [1.3%]), *M. intracellulare* (culture 7 [6.4%]), *M. elephantis* (culture 7 [70.3%]), *M. holsaticum* (cultures 8 [99.4%], 20 [0.7%]), *M. gallinarum* (culture 16 [100%]), *M. brumae* (culture 17 [97.3%]), and *M. alvei* (culture 22 [94.4%]) (Table 2). Nine isolates displayed NTM mixtures with *hsp65* alone (cultures 3,6,7,8,17,20,21,22,27), with four indicating co-infection involving NTM and MTBC (cultures 3,7,21,27), as revealed by the *hsp65* amplicon sequencing analysis. The *gyrB2* gene target successfully identified MTBC in culture 26, whereas *gyrA, gyrB1*, and MAC*hsp65* did not yield any relevant sequences. The five MTBC cultures identified with tNGS ONT sequencing were not from the same patients who had recent pulmonary MTBC detected on sputum GXPU (Table 1). The *rpoB* gene identified *M. monacense* (cultures 2 [0.8%], 4 [100%], 11 [100%], 12 [100%], 14 [100%], 25 [100%]), *M. duvalii* (cultures 3 [100%], 13 [72.6%], *M. elephantis* (cultures 5 [100%], 7 [100%], 10 [1.5%], 20 [94%]), *M. holsaticum* (cultures 8 [98.9%], 10 [1.4%]), *M. brumae* (culture 17 [100%]), *M. neumannii* (cultures 13 [27.4%], 19 [100%], 21 [0.1%]), and *M. avium* (culture 23 [96.5%]) (Table 2). Seven of the cultures depicted an NTM mixture based on *rpoB* gene sequencing (cultures 2, 8, 10, 13, 20, 21, 23). Among the 28 specimens, 19 (68%) exhibited a mixture with identification discrepancies between the *hsp65* and *rpoB* characterizations (Table 1). In addition, four of 28 (14%) displayed uncharacterized mycobacteria species on *hsp65* and *rpoB* sequences (cultures 1, 15, 18, 26) (Table 1). *M. monacense* was present in almost half of the cultures (46%; 13 of 28 isolates). The negative control did not produce reads. The comparison between Sanger sequencing and tNGS ONT sequencing for the *hsp65* gene showed an agreement in 27 of 28 (96%) of the cultures when specifically assessing the predominant NTM strain. A discordant result was observed for culture 23 (Table 1); Sanger sequencing identified *M. avium*, whereas tNGS ONT sequencing reported *Mycobacterium* spp. However, the complementary *rpoB* tNGS ONT sequencing detected the *M. avium* as the prominent strain, alongside a *Mycobacterium* spp*.*

**Table 2:** Identification (proportion percentages) of mycobacteria species using Oxford nanopore technology deep sequence targeting of the heat-shock protein 65 (*hsp65*) and RNA polymerase beta subunit (*rpoB*) gene with Deeplex Myc-TB analysis on selected cultures.

	Mycobacteria																											
Isolate	1	2	3	4	5	6	7	8	9	10	11	12	13	14	15	16	17	18	19	20	21	22	23	24	25	26	27	28
Heat-shock protein 65 (*hsp65*)																												
*M. novocastrense*													100						100	0.3	98.1							
*Mycobacterium* spp*.*	100				100	3.9	16.5			100					100			100		1.2		1.8	100			100	99.1	
*M. monacense*		100	1.8	100					100		100	100		100			2.3			1.2		3.8		100	100			100
*M. duvalii*			97.2																									
*M. tuberculosis* complex			1				5.5														0.3						0.9	
*M. malmesburyense*						96.1		0.6												96.6								
*M. senuense*																					1.6							
*M. abscessus* subsp*. bolletii*							1.3																					
*M. intracellulare*							6.4																					
*M. elephantis*							70.3																					
*M. holsaticum*								99.4												0.7								
*M. gallinarum*																100												
*M. brumae*																	97.3											
*M. alvei*																						94.4						

**Total number of reads for target**	**93208**	**68104**	**100- 318**	**108- 596**	**113- 629**	**65892**	**54797**	**118- 105**	**103- 414**	**57179**	**109- 750**	**65426**	**69799**	**58445**	**55508**	**63691**	**42763**	**110- 437**	**107- 821**	**45775**	**76851**	**9096**	**13336**	**63724**	**85286**	**105- 388**	**54174**	**98502**

**Unknown *Mycobacterium* spp. identifier on *hsp65* phylogenetic tree**	**1H.1**				**5H.1**	**6H.1**	**7H.1**			**10H.1**					**15H.1**			**18H.1**		**20H.1**		**22H.1-2**^a^	**23H.1**			**26H.1**	**27H.2**^a^	

RNA polymerase beta subunit (*rpoB*)																												

*Mycobacterium* spp*.*	100	86.9				100		1.1	100	98.1					100	100		100		6	99.9	100	3.5	100		100	100	100
*M. monacense*		0.8		100							100	100		100											100			
*Unclassified*		12.3																										
*M. duvalii*			100										72.6															
*M. elephantis*					100		100			1.5										94								
*M. holsaticum*								98.9		1.4																		
*M. brumae*																	100											
*M. neumannii*													27.4						100		0.1							
*M. avium*																							96.5					

**Total number of reads for target**	**76389**	**99096**	**68413**	**67834**	**61147**	**89436**	**121- 646**	**38797**	**73500**	**67324**	**63562**	**113- 240**	**100- 202**	**121- 262**	**120- 887**	**54102**	**120- 896**	**71424**	**69172**	**34826**	**87958**	**66208**	**50851**	**116- 366**	**93593**	**39044**	**87334**	**79455**

**Unknown *Mycobacterium* spp. identifier on *rpoB* phylogenetic tree**	**1R.1**	**2R.1**				**6R.1**		**8R.1**	**9R.1**	**10R.1-5**^a^					**15R.1**	**16R.1**		**18R.1**		**20R.1**	**21R.1**	**22R.1**	**23R.1**	**24R.1**		**26R.1**	**27R.1**	**28R.1**

Deeplex Myc-TB analysis on *Mycobacterium tuberculosis* detected cultures																												

**Deeplex Myc-TB** **(% percentage identification)**			**(100) *M. duvalli***				**(98.9) *M. elephantis***														**(87.4) *M. novocastrense*** **(12.4) *M. pulveris***					**(98.5) *M. parraffinum***^b^	**(90.4) *M. monacense*** **(9.5) *M. paraffinum***	

a In mixed bacterial populations with unidentified *Mycobacteria* spp., reads were segregated before the construction of the phylogenetic trees.

b The *gyrB2* gene target successfully identified *Mycobacterium tuberculosis* complex in culture 26.

### Phylogenetic analysis of targeted NGS data

**Figure 2:**
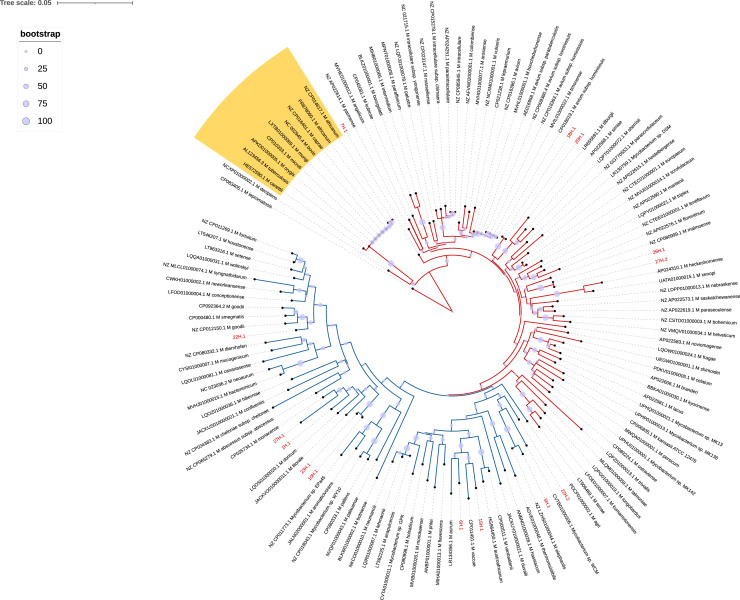
A phylogenetic tree was generated using consensus *hsp65* gene sequences of unidentified mycobacteria species obtained through Oxford Nanopore Technology amplicon-based deep sequencing. The tree illustrates slow-growing mycobacteria represented by red branches and rapid growers indicated by blue branches. Unidentified *Mycobacterium* species sequences from this study are labeled in red. Members of the *Mycobacterium tuberculosis* complex are highlighted in the yellow range. The scale bar expresses the average number of nucleotide substitutions per site. Circular markers signify the bootstrap support of branches within the tree. The isolates included in the construction of the phylogenetic tree are those represented by the reads listed in Table 2: 1H.1, 5H.1, 6H.1, 7H.1, 10H.1, 15H.1, 18H.1, 20H.1, 22H.1, 22H.2, 23H.1, 26H.1, 27H.1, and 27H.2.

**Figure 3:**
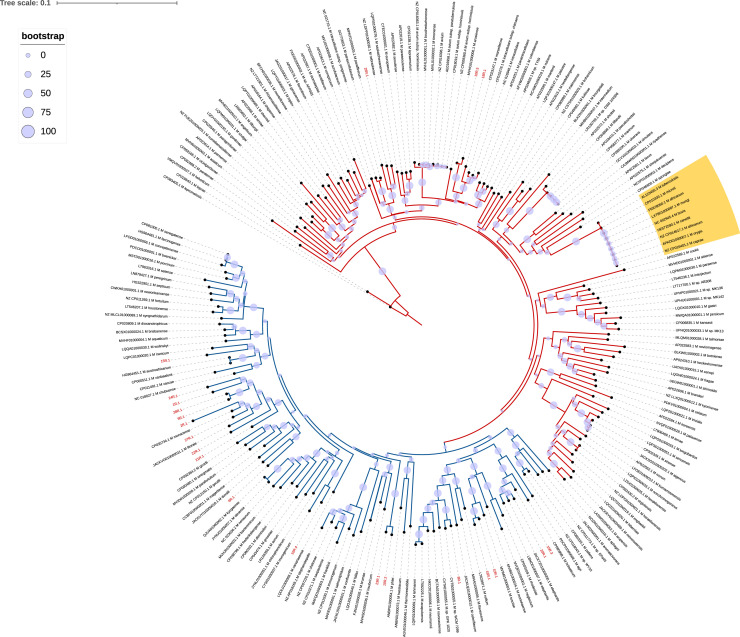
A phylogenetic tree was generated using consensus *rpoB* gene sequences of unidentified mycobacteria species obtained through Oxford Nanopore Technology amplicon-based deep sequencing. The tree illustrates slow-growing mycobacteria represented by red branches and rapid growers indicated by blue branches. Unidentified *Mycobacterium* species sequences from the study are labeled in red. Members of the *Mycobacterium tuberculosis* complex are highlighted in the yellow range. The scale bar expresses the average number of nucleotide substitutions per site. Circular markers signify the bootstrap support of branches within the tree. The isolates included in the construction of the phylogenetic tree are those represented by the reads listed in Table 2: 1R.1, 2R.1, 6R.1, 8R.1, 9R.1, 10R.1, 10R.2, 10R.3, 10R.4, 10R.5, 15R.1, 16R.1, 18R.1, 20R.1, 21R.1, 22R.1, 23R.1, 24R.1, 26R.1, 27R.1, 28R.1.

The *hsp65* phylogenetic tree representing uncharacterized *Mycobacterium* species incorporated five isolates among the slow growers and nine among the fast growers (Figure 2). In contrast, the *rpoB* tree identified only three isolates among the slow growers, with a predominant representation of 18 mycobacteria sequences among the fast growers (Figure 3).

### Deeplex Myc-TB analysis

The Deeplex Myc-TB run had an average percentage Q30 score of ≥93.5%, with 93.6% of clusters passing the filter test, and a PhiX error rate of 0.37%.; *M. duvalii, M. elephantis, M. novocastrense*/*M. pulveris* mixture, *M. paraffinicum*, and a *M. monacense*/*M. paraffinicum* were identified (Table 2). MTBC was not found in any of the five cultures identified with tNGS ONT deep sequencing. Additional Deeplex Myc-TB analysis detail on average coverage depth, consensus length, expect value, and percentage identification is available in the supplementary material (Table S2).

## Discussion

The incorporation of multiple targets enhances the identification of a broader spectrum of NTM and facilitates the detection of NTM mixtures [12]. In this regard, we were able to identify 11 NTM species which are not present on the GenoType Mycobacterium CM/AS LPAs. In our investigation, we found 19 cultures containing at least two distinct NTM strains using tNGS ONT long-read deep sequencing. In addition, four displayed the simultaneous presence of mycobacterial species which could not be identified with the *rpoB* and *hsp65* tNGS ONT sequencing. This finding suggests the potential existence of novel strains. These findings emphasize the necessity for advanced methodologies capable of comprehensively characterizing complex mycobacterial profiles.

Among all cultures that were GXPU-negative after MGIT culture, we identified five cultures (18%, five of 28) with evidence of a MTBC co-infection on targeted tNGS ONT deep sequencing of the *hsp65* (n = 4) and *gyrB2* (n = 1) genes, highlighting the utility of deep sequencing platforms to detect populations below the limit of detection (LOD) of commonly used practices in diagnostic laboratories. In this context, GXPU has an LOD of 15.6 bacterial colony-forming units/ml [24]. Likewise, Deeplex Myc-TB was unable to identify these minor subpopulations that were mostly <3% of the reads for *hsp65* (3% LOD for Deeplex Myc-TB) [15].

Through tNGS ONT, we identified a high prevalence of the NTM strain *M. monacense* (46.4%, 13 of 28 isolates) in cultures obtained from extrapulmonary clinical sites. This finding aligns with a separate study performed in Saudi Arabia that evaluated the 16S rRNA, *rpoB, hsp65*, and 16-23S internal transcribed spacer region genes of 27 LPA unidentified mycobacteria species [8]. The authors identified 16 distinct NTM species among the clinical isolates, with *M. monacense* being the most prevalent. In other parts of the world, such as Brazil, *M. monacense* has also been found among sterile sites [25]. The consistent identification of *M. monacense* from sterile and extrapulmonary sites points to its possible significance in the clinical setting.

The comparison between Sanger and tNGS ONT sequencing showed an 96% agreement when specifically assessing the predominant NTM strain within each culture, focusing on the *hsp65* target. This suggests good agreement between the first-generation sequencing and NGS methods in identifying the prevalent NTM strain present in the analyzed cultures with the target tested. Although Sanger sequencing is regarded as the gold standard due to its low error rate and high accuracy, it is restricted to distinguish between mixed populations [26]. The current tNGS ONT database is curated, in contrast to the basic alignment tool used to interpret Sanger sequencing, which can account for discordant results. Due to the cost-effectiveness and easy workflow of Sanger sequencing of a single gene, it emerges as an appealing choice for NTM identification when using pure cultures [1]. It could be considered as an alternative to tNGS ONT if future clinical studies find that targeting the predominant strain to be the most important factor from a treatment perspective and if stringent identification criterion are met [1].

The consistent identification of mixed NTM infections in the current study is unexpected. The existing literature does not provide a clear understanding of why NTM mixtures occur in cases of extrapulmonary clinical sites. A recent study has suggested that different ectatic bronchi or nodules may harbor different NTM at the initial diagnosis of pulmonary disease [27]. The number of initially detected major NTM species was hypothesized to be minimized with treatment, whereas minor NTM species prevail and emerge at a later stage [28]. Similarly, NTM biofilm formation in extrapulmonary sites could potentially house NTM communities with different population frequencies [28]. However, the relationship between different NTM strains and the potential underlying physiological interactions in NTM communities remain elusive. This highlights the need for further in-depth research to unravel the intricacies of NTM mixtures, especially in extrapulmonary clinical sites. The ability of NTM to survive and replicate within macrophages contributes to their pathogenicity and ability to cause chronic infections. In this sense, macrophages could serve as a reservoir and a means of dissemination for NTM within the body, contributing to the spread of infection to different tissues and organs [29]. Studies on the interaction between NTM and macrophages remain an area to explore. Moreover, determining the phylogenetic relatedness of unknown species to closely identified NTM can offer insights into potential growth features (slow- or fast-growing), biochemistry characteristics, and possible shared virulence factors, aiding in the classification and targeted study of unidentified NTM [29,30]. This approach might narrow the research focus to enhance our understanding and classification of these unidentified mycobacteria that requires thorough phenotypic characterization and drug susceptibility testing to achieve a comprehensive analysis.

The 1-year all-cause mortality of patients in this study was high at 25%, compared with a retrospective study analyzing data from hospitalized patients in the United States, that found a crude overall mortality rate of 5% for extrapulmonary sites, mainly incorporating LPA-confirmed cultures [31]. This suggests the possible clinical importance of these mycobacterial mixtures or the strains identified in this study. The predominance of slow growers among the NTM mixtures is evident, with an average time to MGIT culture positivity of almost 19 days. This contrasts with the phylogenetic analysis of the potential novel species, which were mostly fast growers. With some paucibacillary cultures flagging positive only after 40 days, it is advisable for laboratories not to consider shortening incubation periods when NTM infection is suspected.

A statement from the 2007 American Thoracic Society and the Infectious Diseases Society of America on the diagnosis, treatment, and prevention of NTM diseases expresses the uncertainty of clinical significance and treatment recommendation when interpreting co-infections involving more than one mycobacterial species [32]. It highlights the importance of careful evaluation of such cases on an individual basis by clinicians and suggests considering expert consultation. Given the advancements in culture-independent diagnostics, clinicians are urged to utilize all available clinical and laboratory data to make accurate diagnostic decisions, even from extrapulmonary sites.

We acknowledge limitations to this study. First, it is important to note that the culture cohort is small. Nevertheless, this culture collection represents a rare group that has been gathered over an extended number of years and geographic location (Figure S3), which contributes to the uniqueness and value of the data, providing insights into a distinctive subset of cases that may not be easily accessible for research purposes. Second, because this was a retrospective study, we were not able to collect treatment data, including regimens and therapy durations on these NTM mixed cultures. This should form part of prospective longitudinal studies to comprehensively determine the drug treatment factors influencing outcomes. Third, the use of a culture-independent method directly from the original specimen submitted could have reflected the true composition of mycobacteria in the cultures more closely. However, these specimens were not available. Finally, the study lacks an algorithm for guiding the recommendation of clinical cases that would benefit from tNGS ONT amplicon-based long-read deep sequencing to identify potential NTM subpopulations. This algorithm would likely focus on sterile sites where laboratory culture conversion or clinical response is not evident. The absence of such guidance limits the practical application of tNGS ONT sequencing, emphasizing the need for future research to develop targeted algorithms for its effective utilization, especially in low- to middle-income countries with limited resources.

## Conclusion

ONT amplicon-based deep sequencing revealed NTM-MTBC co-infections and NTM mixtures in MGIT cultures from extrapulmonary samples. Whether detecting these underlying populations will enhance clinical outcomes or complicate matters for clinicians, potentially restricting antibiotic choices, remains an area needing further clarification. The high *M. monacense* prevalence among extrapulmonary cultures suggests its clinical relevance, warranting further investigation. Although comparative analyses show good agreement between Sanger sequencing and tNGS ONT sequencing for detecting prominent strains within NTM mixtures, it is essential to obtain a pure culture for accurate identification and analysis. In addition, research should concentrate on examining the effects of mixed mycobacterial infections, particularly, those involving a combination of slow-growing and fast-growing mycobacteria, on patient management, outcome, and diagnostic practices. Lastly, this study highlights that the integration of targeted NGS into testing algorithms for mycobacteria identification has the potential to enhance diagnostic accuracy and should be the focus of future research endeavors.

## Declarations of competing interest

The authors have declared that no competing interest exists.

## Appendix Supplementary materials

Image, application 1
