# Supplementary material for: Targeted deep sequencing of mycobacteria species from extrapulmonary sites not identified by routine line probe assays: A retrospective laboratory analysis of stored clinical cultures
Source: IJID Reg. 2024 Sep 24;13:100464. doi: 10.1016/j.ijregi.2024.100464 (PMC11526053; doi:10.1016/j.ijregi.2024.100464)
Supplement: Supplementary file 1 [file mmc1.docx]

**Targeted deep sequencing of mycobacteria species from extrapulmonary sites not identified by routine line probe assays: a retrospective laboratory analysis of stored clinical cultures**

**Supplementary Material**

| **Table of contents** | **Page number** |
| --- | --- |
| 1. DNA extraction for targeted amplicon-based next generation sequencing using Oxford Nanopore Technologies sequencing, Sanger sequencing, and Deeplex®Myc-TB | 2 |
| 2. Oxford Nanopore Technology targeted deep amplicon-based next generation sequencing | 2-4 |
| 3. Bioinformatics for targeted amplicon-based next generation sequencing using Oxford Nanopore Technologies sequencing | 5-6 |
| 4. Sanger sequencing *hsp65* gene primers and PCR thermocycling conditions | 6-7 |
| 5. Deeplex®Myc-TB analysis | 7-10 |
| Table S1. Primers and thermocycling conditions for targeted amplicon-based sequencing | 11-12 |
| Table S2. Deeplex®Myc-TB analysis of cultures that displayed *Mycobacterium tuberculosis* complex with Oxford Nanopore Technology targeted deep amplicon-based sequencing | 13 |
| Figure S1. Nontuberculous mycobacteria isolated from clinical extrapulmonary site cultures across the Western Cape Province, South Africa, between 1 January 2020 and 31 December 2020 | 14 |
| Figure S2. Distribution analysis of sequencing reads and mean read quality score among clinical extrapulmonary site cultures | 15 |
| Figure S3. Spatial-temporal mapping of cultures collected between 1 January 2019 and 31 December 2023 in the Western Cape Province, South Africa | 16 |

1. DNA extraction for targeted amplicon-based next generation sequencing using Oxford Nanopore Technologies sequencing, Sanger sequencing, and Deeplex®Myc-TB

Total DNA extraction was conducted employing a modified version of the DNeasy Blood and Tissue kit (Qiagen, Hilden, Germany), directly from one milliliters (mL) of Mycobacteria Growth Indicator Tube (MGIT; Becton Dickinson, Berkshire, United Kingdom). In a concise overview of the procedure, following heat-inactivation at 98°C for 45 minutes and subsequent centrifugation at 1 500 x gravity (g) for 10 minutes, 300 microliters (μL) of buffer ATL was introduced to the cellular pellet. Subsequently, 25 μL of proteinase K was incorporated, initiating a digestion period at 55 degrees Celsius (°C) overnight. The resulting solution underwent centrifugation at 5 500 x g for five minutes, with 500 μL of the supernatant retrieved and transferred to a 1.5 mL tube. Following this, 400 μL of buffer AL and 400 μL of ethanol were added to the solution, and the resultant mixture was transferred to a Mini Spin, column. Ultimately, the isolated DNA underwent purification utilizing wash buffers AW1 and AW2 and was eluted in 60 μL of AE buffer prewarmed to 54°C.

2. Oxford Nanopore Technology targeted deep amplicon-based next generation sequencing

The PCR master mix was composed of 8.5 μL nuclease-free water, 12.5 μL NEB One Taq® Hot start 2X Master Mix (New England Biolabs, Massachusetts, United States) with standard buffer, one μL each of forward and reverse primers 10 micromolar (μM) [10 picomolar (pmol)/μL], and two μL of extracted DNA. This resulted in a final reaction volume of 25 μL per culture. Thermocycling conditions and specific primer details are outlined in Table S1.

For native barcoding ligation, 12.5 μL of 300 femtomol combined polymerase chain reaction (PCR) amplicons per each culture (confirmed via gel electrophoresis) were added to 1.75 μL NEB Ultra II End Prep Reaction buffer (New England Biolabs, Massachusetts, United States) with 0.75 μL Ultra II End Prep Enzyme Mix (New England Biolabs, Massachusetts, United States). The end-repaired DNA mixture was pipetted and incubated at 20°C for five minutes. To prepare the DNA barcoding reaction, three μL nuclease-free water, 0.75 μL end-repaired DNA, 1.25 μL unique native barcode, and five μL NEB Blunt/TA ligase Master Mix (New England Biolabs, Massachusetts, United States) were combined for each culture. Barcoded cultures were incubated for 20 minutes at room temperature. To stop the barcoding reaction, one μL ethylenediaminetetraacetic acid (EDTA) was added to each well. Barcoded cultures (11 μL) were pooled in a 1.5 mL low-bind microcentrifuge tube. AMPure XP beads (Beckman-Coulter, California, United States of America) were added at 0.4X the pooled reaction volume and incubated for 10 minutes at room temperature on a mixer. The culture and pellet were then spun down on a magnet for five minutes until the eluate was clear. The supernatant was discarded, and the beads were washed with 80% freshly prepared ethanol (700 μL) on the magnetic rack. After removing residual ethanol, the pellet was briefly dried for 30 seconds. The tube was taken off the magnetic rack, and the pellet was resuspended in 35 μL nuclease-free water. The resuspended pellet was incubated for 10 minutes at 37°C with agitation every two minutes. Subsequently, the tube was placed back on the magnetic rack, and 35 μL of clear eluate was pipetted off into a new 1.5 mL tube.

For adapter ligation, 30 μL of the pooled barcoded culture was combined with five μL of Native adapter, 10 μL NEB Quick Ligation Reaction Buffer (New England Biolabs, Massachusetts, United States), and five μL of Quick T4 DNA Ligase (New England Biolabs, Massachusetts, United States) in a 1.5 mL low-bind microcentrifuge tube. The mixture was incubated for 20 minutes at room temperature. An AMPure XP (Beckman-Coulter, California, United States of America) cleanup was performed as previously described. After an additional 10-minute incubation at room temperature on a mixer, the culture and pellet were centrifuged on a magnet for five minutes until the eluate became clear. The supernatant was discarded, and the beads were washed with short fragment buffer (700 μL) on the magnetic rack. Following the removal of residual short fragment buffer, the pellet was briefly dried for 30 seconds. The pellet was then taken off the magnetic rack and resuspended in 15 μL of elution buffer. The resuspended pellet was incubated for 10 minutes at 37°C with agitation every two minutes. Subsequently, the tube was placed back on the magnetic rack, and 15 μL of clear eluate was pipetted into a new 1.5 mL tube. Finally, the cleaned library was quantified using Qubit double-stranded (ds) DNA High Sensitivity (HS) Assay kit (Life Technologies, California, United States of America) and adjusted to at least 12 μL with a concentration of 10-20 femtomole.

To prime and load the flow cell, the process involved loading a total of one ml of flow cell priming mix [five μL of BSA (50mg/ml), 30 μL of flow cell tether, and 1170 μL of flow cell flush] into the priming port of the flow cell. Lastly, 75 μL of the prepared library (comprising 37.5 μL sequencing buffer, 25.5 μL library beads, and 12 μL DNA library) was added to the flow cell. The sequencing was carried out using the MinION mk1C device (ONT, Oxford, United Kingdom).

3. Bioinformatics for targeted amplicon-based next generation sequencing using Oxford Nanopore Technologies sequencing

Bioinformatics analysis followed the approach outlined by Ghielmetti et al., 2023 [1]. In summary, 28 datasets generated by sequencing carried out using the MinION mk1C device (ONT, Oxford, United Kingdom) were subjected to analysis. Base calling (260bps – High-Accuracy), de-multiplexing, and barcode trimming were performed using Guppy [v6.4.6] after exceeding 24 hours of data acquisition. The quality assessment of sequencing reads involved the use of FastQC (v0.11.9) and pycoQC (v2.5.0.23). Nanoq (v 0.10.0) was used for filtering reads with a Q score below 12 and generating summary reports. Thereafter, reference-free read sorting was performed using the amplicon sorter tool version 2023-06-19 [2]. A total of 200 000 randomly chosen reads with lengths of between 50-2000 base pairs (bp) were selected for each barcode. Consensus sequences were grouped based on amplicon size and genetic similarity, and relative abundancies retrieved based on the representative pool of reads analysed. The ABRicate software tool was applied for the screening of consensus sequences against customized databases for each target and generate summarizing report files [1,3]. Consensus sequences with identity match <90% were annotated as unclassified. Consensus sequences with coverage >90% and identity match between 90-98% were classified to genus level. Consensus sequences with coverage >90% and an identity match >98% were assigned the mycobacterial species or complex with the highest identity match. For clarity purposes, the mycobacterial genus discussed in this manuscript was referred to as *Mycobacterium*, disregarding the recent proposed nomenclature introducing four new genera (*Mycoabacteroides*, *Mycolicibacter*, *Mycolicibacterium*, and *Mycolicibacillus*). The phylogeny and taxonomy of these clades are still being debated [4].

**Bioinformatics references:**

[1] Ghielmetti G, Loubser J, Kerr TJ, Stuber T, Thacker T, Martin LC, et al. Advancing animal tuberculosis surveillance using culture-independent long-read whole-genome sequencing. Front Microbiol 2023;14:1307440. doi: 10.3389/fmicb.2023.1307440.

[2] Vierstraete AR, Braeckman BP. Amplicon_sorter: A tool for reference-free amplicon sorting based on sequence similarity and for building consensus sequences. Ecol Evol 2022;12(3):e8603. doi: 10.1002/ece3.8603.

[3] Torsten S. ABRicate, https://github.com/tseemann/abricate [Accessed 1 February 2024).

[4 Ghielmetti G, Giger U. *Mycobacterium avium*: an emerging pathogen for dog breeds with hereditary immunodeficiencies. Curr Clin Microbiol Rep 2020;7(3):67-80. doi: 10.1007/s40588-020-00145-5.

3. Sanger sequencing *hsp65* gene primers and PCR thermocyler conditions

The heat shock protein 65 (*hsp65*) gene primers, forward 5’-ACCAACGATGGTGTGTCCAT-3’ and reverse 5’-CTTGTCGAACCGCATACCCT-3’, were utilized. Each 25 μL PCR reaction mixture comprised 12.5 μL NEB One Taq® Hot start 2X Master Mix (New England Biolabs, Massachusetts, United States), one μL from each 50 μM primer stock solution, 5.5 μL sterile nuclease-free water, and five μL of undiluted extracted DNA. The thermal cycling protocol initiated with an initial denaturation step at 94°C for 10 minutes. Subsequently, 40 cycles were performed, involving denaturation at 94°C for 30 seconds, annealing at 62.5°C for 30 seconds, and elongation at 72°C for 30seconds, followed by a final elongation at 72°C for 5 minutes, and a 4°C hold. The confirmation of amplified product target size was accomplished through 1.5% agarose gel electrophoresis. Subsequent visualization and documentation were facilitated by the ChemiDoc M.D. Universal Hood III Gel Documentation System (Bio-Rad Laboratories, Hercules, California, USA).

4. Deeplex®Myc-TB analysis

The Deeplex®Myc-TB master mix (Genoscreen, Lille, France), consisting of 15.8 µl, was added to 0.2 µl of a diluted internal control, serving as a non-mycobacterial DNA control for PCR inhibition. This mixture was distributed across all wells, excluding the last designated for the negative control, which only contained the master mix. For each isolate, nine µl of DNA suspension (>one pg/µl) was added to the respective wells, and the positive control well received nine µl of diluted *Mycobacterium bovis* solution. PCR conditions involved preheating the lid to 100°C, setting the reaction volume to 25 µl, and implementing a cycling protocol of 98°C for two minutes, followed by 35 cycles of 94°C for one minute, 55°C for 30 seconds, 72°C for one minute and 30 seconds, and a final extension at 72°C for 10 minutes with a hold at 4°C.

The subsequent steps included an amplicon clean-up process, starting with the addition of 75 µl of 10 mM Tris-HCl pH 7.8 to each well, along with 65 µl of Agencourt AMPure XP® (Beckman-Coulter, California, United States of America) bead suspension. After room temperature incubation and magnetic rack placement, supernatant was discarded, and two washing steps with freshly prepared 80% ethanol were performed. Residual ethanol traces were removed, and the beads were allowed to dry for approximately 10 minutes. Following resuspension in 26 µl of 10 mM Tris-HCl pH 7.8, the beads underwent further incubation and magnetic separation before transferring 25 µl of purified amplicon DNA to a new plate. Confirmation of the quantification of purified amplification products involved ensuring high-quality DNA (>10 µl, 260/280 ratio of 1.8) at a concentration above one ng/µl.

The Qubit double-stranded (ds) DNA High Sensitivity (HS) Assay kit (Life Technologies, California, United States of America) assay, encompassing both broad-range (for genomic DNA quantitation) and high sensitivity (for library quantitation), included stringent verification steps. The negative control was ensured to be below the detection level, and the external positive control exhibited a concentration well above 0.2 ng/µl. The concentration of the cultures tested was ideally maintained at a minimum of 0.2 ng/µl.

Library preparation involved the utilization of five µl of input DNA at 20 ng/µl, with the addition of 20 µl of tagmentation master mix to each culture well containing a 100 ng DNA dilution. Running the cultures on a thermocycler followed a specific program (preheat lid to 100°C, reaction volume to 50 µl, 55°C for 15 min, hold at 10°C).  During post-tagmentation clean-up, 10 µl of tagment stop buffer (TSB) was added to each well and resuspended. PCR conditions involved preheating the lid to 100°C, setting the reaction volume to 60 µl, incubating at 37°C for 15 minutes, and holding at 4°C. Subsequently, tubes were placed in a magnetic plate, allowing the liquid to clear (~3 minutes). The supernatant was discarded, and the beads underwent three washes with 100 µl of tagment wash buffer (TWB). To amplify the tagmented DNA, 40 µl of PCR master mix was directly added onto the beads in each culture. The selected cycling thresholds involved reheating the lid to 100°C, selecting a reaction volume of 50 µl, 68°C for three minutes, 98°C for three minutes, followed by cycles of 98°C for 45 seconds, 62°C for 30 seconds, 68°C for two minutes, 68°C for one minute, and holding at 10°C.

Cleaning the libraries included centrifuging the strip tubes at 280 X g for one minute, using the magnetic stand to ensure clear liquid. Subsequently, 45 µl of supernatant was transferred, and 40 µl of nuclease-free water was added along with 45 µl of culture purification beads (SPB) and resuspended. This mixture incubated for five minutes at room temperature, and the tubes were placed on a magnetic bead until the liquid was clear. Transferring 125 µl of supernatant to a clear plate along with 15 µl of SPB followed, with another five-minute incubation and magnetic stand usage. Discarding the supernatant and performing two washes with 200 µl of 80% ethanol were part of the subsequent steps. After removing residual ethanol and air-drying for five minutes, 32 µl of resuspension buffer was added. Incubating at room temperature for two minutes and placing on a magnetic stand facilitated the transfer of 30 µl of supernatant to a new plate.

The library was quantified using the dsDNA HS Qubit Assay to achieve a final loading concentration of 1.2 pM. For each culture, a final concentration of two nM in a final solution of 10 µl was obtained. For libraries with a concentration equal to or lower than two nM, 10 µl of undiluted library was used. Pooling calculated volumes for individual libraries into a single tube, a volume of Tris HCl (Merck, Darmstadt, Germany) 10 mM + 0.1% Tween 20 or RSB was added to reach the required total volume equal to the number of cultures x 10 µl. The library pool was kept on ice. The PhiX was denatured by adding five µl of 0.1 N NaOH (Merck, Darmstadt, Germany Merck) in an Eppendorf lo-binding tube, containing five µl of four nM PhiX. This was briefly vortexed and pulse centrifuged, incubated at room temperature for 5 minutes, and 5 µl of 200 mM Tris-HCl, pH 7.0 (Thermo Fisher Scientific, Waltham, Massachusetts, United Sates of America) added. To dilute the denatured PhiX to a loading concentration, 985 µl of pre-chilled HT1 was added to the denatured PhiX for a total volume of one ml at 20 pM. Finally, the loading volume for the MiniSeq System was 500 µl at a loading concentration of 1.2 pM and a 1% PhiX spike. The flow cell was thawed for 30 minutes at room temperature before use.

Table S1. Primers and thermocycling conditions for targeted amplicon-based sequencing.

| **Name** | **Forward/**  **Reverse** | **Sequence 5'-3' [1-4]** | **Size (bp)** | **Annealing temperature^a^ (℃)** | **Elongation time^b^** |
| --- | --- | --- | --- | --- | --- |
| *hsp65* | Forward | ACCAACGATGGTGTGTCCAT | 441 | 62.5 | 30 seconds |
|  | Reverse | CTTGTCGAACCGCATACCCT |  |  |  |
| *MAChsp65* | Forward | AATTGCGTACGACGAAGAGG | 1621 | 55 | 2 minutes |
|  | Reverse | ACGGACTCAGAAGTCCATGC |  |  |  |
| *rpoB* | Forward | GGCAAGGTCACCCCGAAGGG | 764 | 64 | 1 minute |
|  | Reverse | AGCGGCTGCTGGGTGATCATC |  |  |  |
| *GyrB1* | Forward | CGGCTCGAAGTCGAGATCAAG | 144 | 55 | 30 seconds |
|  | Reverse | TTCGAAAACAGCGGGGTCG |  |  |  |
| *GyrB2* | Forward | CAAATCGTTTGTGCAGAAGGTCTG | 107 | 55 | 30 seconds |
|  | Reverse | CTTGCGCCGAGGACACAG |  |  |  |
| *GyrA* | Forward | AGGCAATCCTGGACATGCAG | 107 | 55 | 30 seconds |
|  | Reverse | GATGTCTTCCAGATCGGCGATC |  |  |  |

Denaturation was carried out at 94°C for 15 minutes for 1 cycle, followed by 35 cycles of denaturation at 94°C for 30 seconds, annealing at variable temperatures^a^ for 30 seconds, and elongation at 68°C for variable durations^b^. A final elongation cycle was performed at 72°C for 5 minutes with a hold at 4°C.

**Primer References:**

[1] Ghielmetti G, Loubser J, Kerr TJ, Stuber T, Thacker T, Martin LC, et al. Advancing animal tuberculosis surveillance using culture-independent long-read whole-genome sequencing. Front Microbiol 2023;14:1307440. doi: 10.3389/fmicb.2023.1307440.

[2] Adékambi T, Colson P, Drancourt M. *rpo*B-based identification of nonpigmented and late-pigmenting rapidly growing mycobacteria. J Clin Microbiol 2003;41(12):5699-708. doi: 10.1128/JCM.41.12.5699-5708.2003.

[3] Turenne CY, Semret M, Cousins DV, Collins DM, Behr MA. Sequencing of *hsp*65 distinguishes among subsets of the *Mycobacterium avium* complex. J Clin Microbiol 2006;44(2):433-40. doi: 10.1128/JCM.44.2.433-440.2006.

[4] Landolt P, Stephan R, Stevens MJA, Scherrer S. Three-reaction high-resolution melting assay for rapid differentiation of *Mycobacterium tuberculosis* complex members. Microbiologyopen 2019;8(12):e919. doi: 10.1002/mbo3.919.

Table S2. Deeplex®Myc-TB analysis of cultures that displayed *Mycobacterium tuberculosis* complex with Oxford Nanopore Technology targeted deep amplicon-based sequencing.

| Patient culture | Mycobacteria species | Average coverage depth | Consensus length (base pairs) | Expect value | Percentage identification (%) |
| --- | --- | --- | --- | --- | --- |
| 3 | *Mycobacterium duvalii* | 4359.3 | 400 | 0.0 | 100% |
| 7 | *Mycobacterium elephantis* | 8.3 | 398 | 0.0 | 98.9% |
| 21 | *Mycobacterium novocastrense/ Mycobacterium pulveris* | 848.1 | 400 | 0.0/1.8e-180 | 87.4%/12.4% |
| 26 | *Mycobacterium paraffinicum* | 225910.2 | 400 | 0.0 | 98.5 |
| 27 | *Mycobacterium monacense/ Mycobacterium paraffinicum* | 776.9 | 400 | 0.0 | 90.4%/9.5% |

Figure S1. Nontuberculous mycobacteria isolated from clinical extrapulmonary site cultures across the Western Cape Province, South Africa, between 1 January 2020 and 31 December 2020. The nontuberculous mycobacteria were identified from Mycobacterial Growth Indicator Tube (MGIT) cultures using the GenoType® Mycobacterium Common Mycobacteria (CM) and Additional Species (AS) line probe assays. Fluid aspirates represent a range of extrapulmonary collection sites, including peritoneal, synovial, and pleural spaces, among others.

Figure S2. Distribution analysis of sequencing reads and mean read quality score among clinical extrapulmonary site cultures. All isolates exhibited a sequencing depth exceeding 100 000 reads, accompanied by a mean read quality score greater than 14. Out of these, 200 000 reads (where available) were randomly chosen for subsequent analysis using the amplicon sorter pipeline. Not included in the figure is the reads of *GyrA*, *GyrB1*, and *GyrB2*.


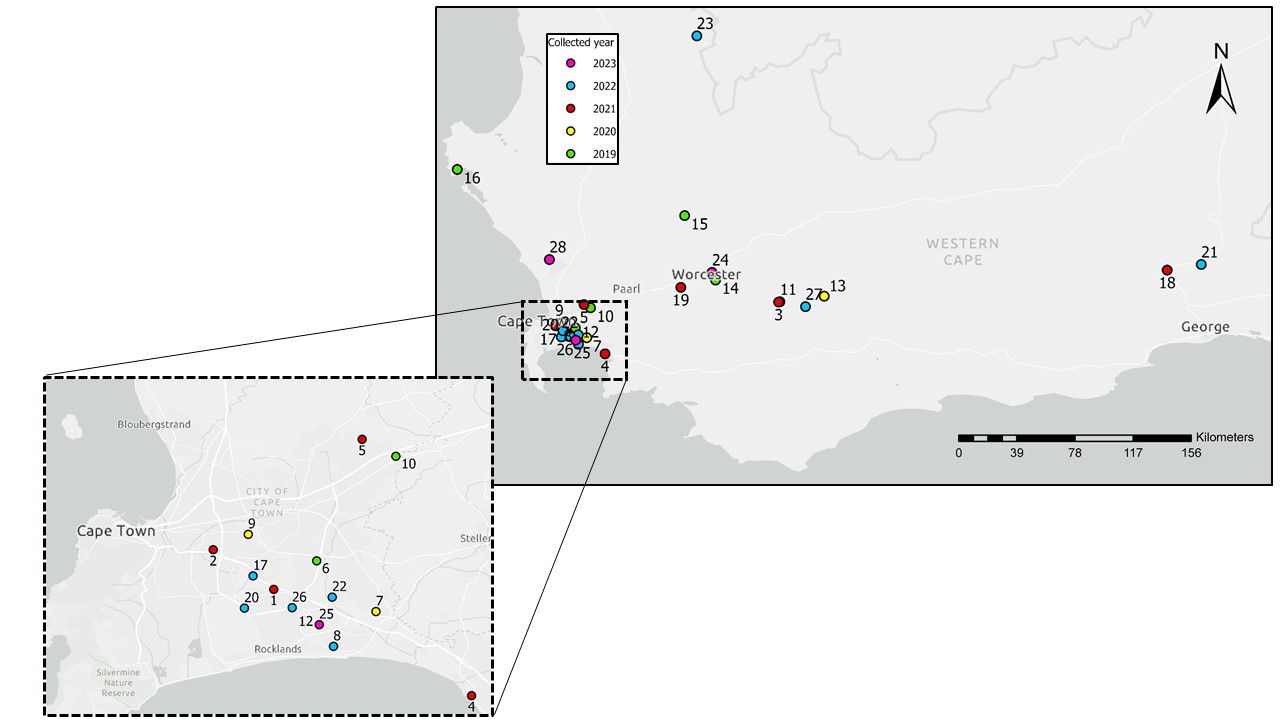


Figure S3. Spatial-temporal mapping of cultures collected between 1 January 2019 and 31 December 2023 in the Western Cape Province, South Africa. Twenty-eight extrapulmonary site cultures were included in the targeted amplicon-based deep sequencing (Oxford Nanopore Technology) analysis of unidentified mycobacteria species according to the GenoType®Mycobacterium Common *Mycobacteria* (CM) and Additional species (AS) line probe assays. Geographical distribution illustrates a wide sampling area, with a notable concentration in the Cape Metropolitan area of Cape Town.
